# The Physiology of Digestion in Ruminants, with Practical Remarks

**Published:** 1882-07

**Authors:** Henry C. Slee


### ART. XVI.—THE PHYSIOLOGY OF DIGESTION IN RUMINANTS, WITH PRACTICAL REMARKS.

(*Concluded.*)

BY HENRY C. SLEE.

### THE LIVER.

The liver in ruminants differs little from that of the horse, the principal distinction being that it is confined to the right side.

The gall-bladder is present in the antelope and the hollow-horned ruminants, but not in the camel or deer. The giraffe has been found with a gall-bladder divided so as to form almost two distinct bladders, but most giraffes have none at all. The gall-bladder is unaccountably capricious, appears in some animals with simple stomachs, while some with complex stomachs have none.

In some ruminants the bile and pancreatic juice reach the small intestine by separate ducts, as in the cow, in which the bile duct is 25 to 30 inches from the pylorus, and the pancreatic duct some 15 inches lower; in the sheep, goat and giraffe, the ducts join and form one which reaches the intestine 12 to 16 inches from the pylorus.

### WATER.

When water is drank the lips of the œsophageal gutter are partially opened and the water goes into the rumen and reticulum, and can be regurgitated in some ruminants without carrying the food with it. The camel, when going long distances, subsists partially upon the fat in his hump. He therefore has no need of water to dissolve or dilute food, and requires only sufficient to moisten his tissues; and he is very economical, even having a duct conveying into his mouth the watery discharges of the nose. He regurgitates the water, which is then caught by the palatal protuberance, and bathes the throat and the root of the tongue. The stag drinks a very small amount of water, sometimes not touching it for two or three months, and to the reindeer water is as great a rarity.

### THE DIGESTIVE JUICES.

The *saliva* of ruminants has no ptyalin (which is the ferment having the power of turning into sugar starchy foods in the saliva of man and the horse), hence its only function is to assist in the lubrication of the food and its conveyance into the actively digestive organs; but it is secreted in enormous quantities, a cow secreting an average of 120 pounds in twenty-four hours, while the horse secretes about 80 pounds, which is large compared with the 2 pounds secreted by a man in the same time. This amount varies with the dryness of the food. A pound of dry hay requires 4 pounds of saliva; wet hay, 3 pounds; barley, oats, and similar grains, 2 pounds; while wet meal is swallowed with a small amount of saliva. During the first mastication the amount of saliva secreted is comparatively small, the food being swallowed without a pause in the process of grazing, and passing down the œsophagus in rather dry, hard masses. These striking against the lips of the "gutter," open them and fall into the rumen. Here it is subjected to constant motion, the rumen moving up and down and forwards and backwards with a kind of "balancing" movement. The papillæ which line the rumen assist the food in its passage through the different compartments, and prevent its moving the other way, as they are erectile and pointing in the direction the food should take. There is no secretion of fluid in this stomach, the food being moistened with water which is taken into the rumen directly by drinking, or indirectly from the cells of the reticulum. There is a considerable rise of temperature in the rumen when it contains food—amounting to 104° F., and a species of fermentation takes place, by which cellulose is partially digested.

When the rumen is full—which, in the cow, requires, on the average, about three hours—the animal seeks a favorable spot in which to lie down and

"RUMINATE."

The process is much easier in this postion than while standing, as the food is pressed up toward the œsophagus. The animal now, by a contraction of the walls of the rumen, which we have seen are movable at will, forces the food past the semilunar valve into the reticulum. And here it has been asserted that the food is formed into pellets in the cells; but there are many reasons why this theory should be doubted, some of which are that the camel, in which these cells are most highly developed, and in the stag, the procese of rumination is much more difficult, apparently, and accomplished with much more exertion than in the goat, in which these cells are less marked; while the giraffe and musk ox have no cells in the reticulum. More probably the reticulum is contracted so as to force the food between the lips of the gutter, which contract both lengthwise and circularly upon a portion of the food; and then, by the motion of the œsophageal muscles, assisted by the abdominal muscles and a deep expiratory effort (similar to the process of vomiting in the human), the pellet is thrown into the mouth with considerable force, the large papillæ in the cheeks assisting in catching it. The moment it arrives there, the fluid portion of it is again swallowed, as can be plainly seen in the cow, the process of swallowing always immediately following regurgitation. Very dry food, such as hay, is often regurgitated two or three times before it is sufficiently macerated to pass the gutter, while the greater part of soft mashes is not regurgitated at all; so, by feeding a cow upon such food, we could stop her ruminating almost entirely.

The act of regurgitation seems to be more difficult to some ruminants than to others. In the stag it requires a considerable effort, and in the camel the action of the abdominal muscles seems much greater than in the cow.

Thr cow regurgitates about 2½ ounces of solid food each time, and this is again masticated for about fifty seconds, accompanied by a copious scretion of saliva. In the motion of the jaws during rumination the camel differs from other ruminants—it moves the jaw alternately from left to right, and from right to left, while other ruminants, though moving the jaw both ways, do not alternate, but suit the motion to the position of the food in the mouth. There is so much saliva now mix d with the food that it becomes a semi-liquid mass, and when swallowed again, glides down the œsophagus without opening the lips of the gutter (which are voluntarily tightened at the same time), and is recei ed into the third stomach. Here it is moved about and pressed between the leaves by the muscular motions, pulverized still further, and formed into a pulp. For the proper action of this stomach a large proportion of fluid is necessary, otherwise the constant rubbing, together with the impaction and fermentation of the food, will produce inflammation; it also requires to be in this form to get out of these leaves into the last stomach; hence we can see the necess ty of their being omitted in the camel, who is compelled to be for long periods with a scanty supply of water.

THE GASTRIC JUICE

secreted in the fourth stomach is similar to that in other animals. Gastric juice does not vary, being essentially the same in the shark and man. The action of this juice, being confined to nitrogenous substances, would at first appear of slight consequence in herbivorous animals, but the proportion will be seen by reference to the table below. Ruminants, with the exception of the camel, secrete proportionately more gastric juice than solipeds. A cow secretes about 50 quarts in twenty-four hours, and a horse about 40 quarts.

Ruminants secrete less *bile* than many other herbivorous animals, although much more than carnivora. According to one statement, the amount secreted in one hour is: Horse, 250 to 300 grammes (8-10 oz.); ox, 100 to 120; sheep, 18 to 20. M. Colin gives the amount secreted in twenty-four hours as follows in kilogrammes: Horse, 6; ox, 2.64; sheep, .34. In the bile of ruminants (and all herbivorous animals) the glycocholate of soda is the principal salt, although there is always some of the taurocholate (the salt of the bile of carnivora) present. The color being green in herbivorous animals instead of red, as in the carnivora, is due to the excess of carbon in the blood, which leads to the formation of bile verdin (green pigment) rather than bile rubin.

The *pancreatic juice* of herbivorous animals is weaker than that of carnivora, but its function of emulsifying fats is equally important, for while ordinary meat contains but 15 per cent. of fat, the proportion in vegetable substances will be seen in the table below.

AN ANALYSIS OF THE FÆCES

of animals, when the amount and nature of the food taken is known, shows the amount of the different substances retained in the body, and in this way the following facts have been discovered:

In herbivorous animals the percentage of the food absorbed is less than in carnivorous or omnivorous animals. The percentage digested on an ordinary diet is: Herbivora, 60 per cent.: pig, 80 per cent.; man, on flesh diet, 95 per cent.; on fruit diet, 87 per cent.; carbohydrates, 99 per cent.

An ox fed upon twenty pounds of clover hay per day was found to digest the following percentage of its constituents: Proteid, 50; fat, 45; carbo-hydrates, 70; cellulose, 40.

One fed on the same amount of meadow hay was found to digest the following percentage: Proteid, 60; fats, 40; carbo-hydrates, 65; cellulose, 60.

Of concentrated foods, such as corn, the greatest amount is absorbed.

The digestion of cellulose and coarse fodder (hay, etc.) is more active in ruminants than in the horse, and as cellulose forms 25 per cent. of hay and nearly 50 per cent. of straw, this is an important fact. From 40 to 60 per cent. of it is digested. Outside of the body it has been found that the only things which affect cellulose are strong acids and alkalis. Boiling with strong acids will turn it into sugar, and experiments by MM. Popoff and Hoppe Seyler have shown that in its decomposition it gives rise to a large amount of carbon-dioxyde and carburetted hydrogen; and as the latter is found in considerable quantities in the large intestines of herbivorous animals, it is inferred that cellulose digestion takes place in the cœcum of the horse, and principally in the colon of ruminants, although some is digested in the paunch.

The digestion of coarse fodder is decreased as more concentrated food is mixed with it.

PERCENTAGE OF FAT, NITROGENOUS COMPOUNDS AND CELLULOSE IN VARIOUS FOODS.

| | Fat. | Nitrogenous Matter. | Cellulose. | The balance other Carbo-hydrates. |
|---|---|---|---|---|
| Hay | 3 | 7 to 12 | 25 | |
| Wheat straw | 3 | 3 | 35 to 50 | |
| Pea and bean straw | — | 7 to 10 | — | |
| Tree leaves | — | 20 | — | |
| Turnips | 2 | 15 | 7 | |
| Cabbage | 3 | 30 | 9 | |
| Potatoes | 1 | 9 | 9 | |
| Cauliflower | — | 64 | — | |
| Wheat bran | 4 | 16 | — | |
| Corn | 9 | 12 | 6 | |
| Oats | 7 | 14 to 20 | 2.5 | |
| Wheat | 1.5 | 10 to 20 | 5 | |
| Barley | 2 | 12 to 15 | 3 | |
| Beans, peas, lentils | 2 | 24 to 30 | 10 | |
| Very young peas | — | 48 | — | |
| Acorns | 3 | 15 | 2 | |
| Rye | 3.5 | 12 | 3 | |
| Buckwheat | 0.4 | 10 | 25 | |
| Linseed | 25 | 20 | 8 | |

